# FT Raman spectroscopy in the evaluation of biomarkers of normal and pathological placenta tissue

**DOI:** 10.1007/s11010-019-03536-5

**Published:** 2019-04-19

**Authors:** Anna Pielesz, Rafał Bobiński, Dorota Biniaś, Andrzej Gawłowski, Wioleta Waksmańska, Izabela Ulman-Włodarz, Tomasz Ilczak

**Affiliations:** 1 Faculty of Materials, Civil and Environmental Engineering, University of Bielsko-Biała, Bielsko-Biała, Poland; 2 Faculty of Health Sciences, University of Bielsko-Biała, Bielsko-Biała, Poland; 3 Department Gyneacology, Provincial Specialist Hospital, 102 Eudkacji St., 43-100 Tychy, Poland

**Keywords:** Trophoblast specimens, Biomarkers of regular and pathological tissue, FTR spectra

## Abstract

The basic precondition of proper intrauterine growth is appropriate supply of nutrients transported through placenta. Placenta capacity in the scope of transportation is dependent on transport systems and the structure of the basement membrane and syncytiotrophoblast microvillous membrane. The present pilot study demonstrates preliminary results of the analysis of placenta structure in the course of selected pathologies by FT Raman spectroscopy analysis. The observed changes of the molecular structure in the so-called average spectra, independent of methodical processing, may be an indicator of the efficiency of transportation controlled by syncytiotrophoblast. In particular, an increase in the intensity of dispersion and transfer within the frequency of 3425–3300 cm^−1^ demonstrate the dynamics of the interaction in the scope of hydrogen bonds in healthy tissues. Changes in the molecular structure within the frequency of 950–750 cm^−1^ and conformational changes within disulphide bonds differentiate the healthy tissue from the pathological one. Changes in the molecular structure observed in the FTR spectra are a spectroscopic image of placenta functions in the course of various pathologies. They also document a complex goal of our research that is finding spectroscopic biomarkers of regular and pathological placental tissue.

## Introduction

Preterm births remain an important and still not completely resolved problem of perinatal medicine. Average annual rate of preterm births is 11%, which equals circa 15 million worldwide. Preterm or/and small for gestational age (SGA) neonate or/and intrauterine growth restriction (IUGR) neonate care is a huge economic problem in neonatal intensive care units. Successful treatment in a neonatal unit does not thoroughly eliminate future negative health effects of prematurity or hypotrophy such as diabetes, cardiovascular diseases, or a decrease in the intelligence level assessed later on the basis of the IQ rate [1–3]. The basic precondition of proper intrauterine growth is appropriate supply of nutrients transported through placenta. Placenta capacity in the scope of transportation is, on the other hand, dependent on specialized transport systems and the unique structure of the basement membrane and syncytiotrophoblast microvillous membrane [3–5]. They are extremely complicated structural and metabolic complexes functioning at the junction of the mother’s and child’s organisms, which not only control fetal development but also “program” the child’s vulnerability to diseases that can develop in the adulthood [5]. The course of these processes results from the coincidence of numerous variables which shape the medical reality, and therefore the issues of physiology and pathobiochemistry of many important processes taking place in the placenta, such as, for instance, maternal-placental-fetal fatty acids metabolism [3] remain poorly understood. In these processes, the syncytiotrophoblast tissues, particularly tissue macromolecules, play an extremely significant role. Currently little is known about their molecular and supermolecular structure, and consequently, about their biochemical role. FTIR and FTR spectroscopic techniques are methods of analysis of biopolymers molecular structure changes [6–8], including protein and lipid tissues (the basement and microvillous) of syncytiotrophoblast. The main subject of assessment as regards changes in polymer structure stabilized by hydrogen bonds are the shifts in the scope of hydrogen bonds/interactions and amide functional groups in the function of a progressive pathology. Spectroscopic validation of protein and lipid structure of placenta membrane in the course of Intrauterine Growth Restriction (IUGR) is very informative, which may give basis for the application of early therapy of the developing fetus. Observed changes of molecular structure visible in the FTR spectra may also be indicators of the efficiency of transport controlled by syncytiotrophoblast, and hence they may be the marker of placenta function in the course of various pathologies such as delivery of an intrauterine growth restriction neonate, intrauterine growth restriction in the course of gestosis, delivery of a large for gestational age neonate, or delivery of a full-term healthy neonate.

The purpose of this research was to find spectroscopic biomarkers, the so-called specific ranges of frequency, which will allow for a precise distinction between regular and pathological placental tissue. It is also worth answering the questions of whether spectroscopic identification of proteins/lipids which bind with the membrane in trophoblast villi is possible and if the changes of molecular structure observed in the FTIR and FTR spectra can point to the efficiency of fatty acid transport through placenta membranes.

## Methods

### Abbreviations

AGA, appropriate for gestational age; IUGR, intrauterine growth restriction; IUGR-G, mothers with gestosis who gave birth to babies diagnosed with IUGR; LGA, large for gestational age; SEM, Scanning Electron Microscopy; FTR, Fourier-Transform Raman Spectrometry; LOI, Limiting Oxygen Index.

### Study population

The study population consisted of 27 women who gave birth at the Provincial Specialist Hospital No. 1 in Tychy, Poland. To obtain a homogeneous group of women, the following inclusion criteria were applied: single pregnancy; pregnancy I-III; stable socioeconomic status; living in a highly industrialized urban region; following a typical diet for the Polish population (none of the women accepted into the study were vegetarians or followed any other special diets); granting consent to participate in the study.

The following exclusion criteria were applied: chronic diseases; pathologies during the course of pregnancy, such as gestational high blood-pressure, infections during pregnancy, miscarriages and/or premature birth resulting in the death of the child, developmental anomalies in the fetus; AIDS and sexually transmitted diseases; lack of the mother’s consent to take part in the research program or withdrawal of consent during the study.

Women who participated in the research program were classified into three groups according to the following criteria:

Group AGA (Appropriate for gestational age, *n* = 13): healthy mothers, routine and uneventful pregnancy, full-term delivery healthy neonates (bw 10th–90th percentile);

Group LGA (Large for gestational age, *n* = 3): mothers who gave birth to full-term but large for gestational age neonates (bw > 90th percentile);

Group IUGR (Intrauterine growth restriction, *n* = 8): mothers who gave birth to babies diagnosed with IUGR;

Group IUGR-G (Intrauterine growth restriction with gestosis, *n* = 3): mothers with gestosis who gave birth to babies diagnosed with IUGR.

Women eligible for the study underwent three ultrasound examinations. The first ultrasound was performed between the 12th and 14th weeks of gestation, the second one between the 20th and 22nd weeks, and the third one in the 32nd–33rd week. All the fetuses had normal karyotypes and no malformations at birth. The mothers did not receive any dietary supplementation during pregnancy.

The study was approved by the Bioethics Committee in Bielsko-Biała (No: 2016/02/11/4), which is in accordance with the Helsinki Declaration.

### Scanning electron microscopy analysis

The placentas obtained during the delivery were immediately transported to the laboratory where trophoblast specimens were extracted and subjected to microscopic examinations. A small amount of tissue was extracted from each sample and was shaken in the physiological saline solution for an hour. Rinsed tissue fragments were immersed in 60% ethanol for 15 min and dried in a laboratory oven at 35 °C. Imaging of the surface structure changes of physiological and pathological placenta tissue was conducted by Scanning Electron Microscopy using a JSM-5500LV scanning electron microscope supplied by JEOL. The samples were mounted on aluminum stubs and coated with gold (JFC 1200, JEOL). Detection of secondary (SE) and back-scattered electrons (BSE) was conducted with an accelerating voltage of 10 kV. Microphotographs were taken at magnifications × 5000, × 2500, and × 1000.

### LOI

The obtained flame retardant effect of placentas was evaluated using the method of the limiting oxygen index (LOI). A parameter that characterizes the method is the lowest percentage of oxygen in the mixture with nitrogen, at which the test specimen ignites and burns on its own. The measurements were performed in accordance with PN–ISO 4589 standard.

### Raman spectroscopic analysis

A NICOLET MAGNA-IR 860 spectrometer with FT Raman accessory was used to record the Raman spectra of the samples. The solid samples were then irradiated with a 1064 nm line YAG laser and scattered radiation was collected with 4 cm^−1^ resolution. The spectra of the three repacked subsamples of each individual sample were averaged to that of one spectrum. All the spectra were obtained using a linear baseline and pre-processed with Fourier smoothing (Grams 32 AI software, Galactic Industries; smoothing degree, 50%).

## Results and discussion

The present pilot study demonstrates preliminary results of the analysis of placenta structure in the course of selected pathologies by FT Raman spectroscopy analysis. This research is primarily a source of a number of useful methodical data concerning the appropriate preparation of samples for spectroscopic studies.

**Fig. 1:**
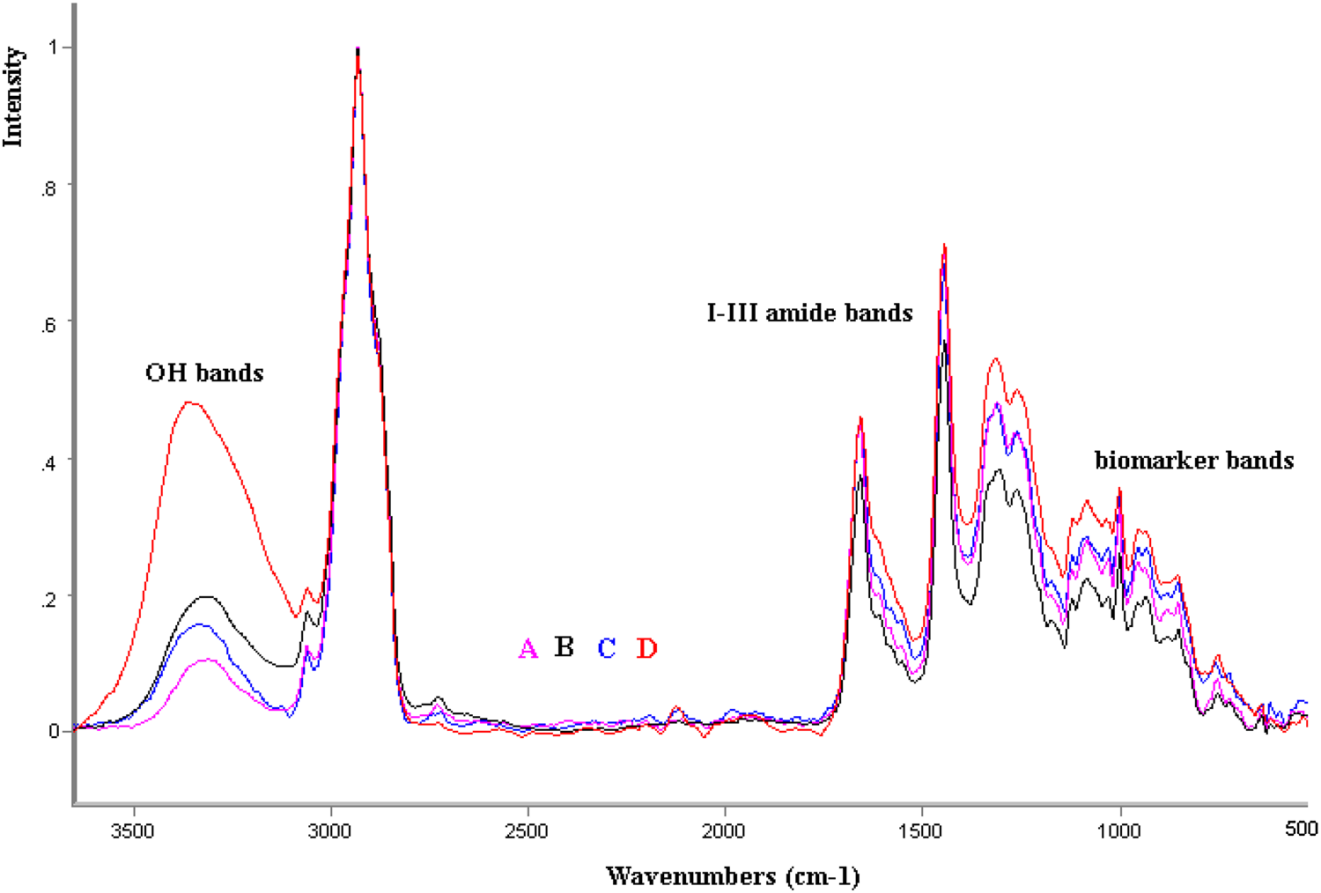
The average FT Raman spectra of the samples: D = AGA-healthy, appropriate for gestational age; A = LGA-healthy, large for gestational age; B = IUGR-intrauterine growth restriction; C = IUGR-G- mothers with gestosis (pregnancy toxemia)

Initially it was observed that the analyzed samples of placenta are characterized by significant variability within frequency in the scope of the so-called fingerprint (1800–1000 cm^−1^) which depends on the process of homogenization, drying, and cleaning of placental tissues. Samples representing average spectra, independent of their methodical processing, were selected for further research in such conditions (Fig. 1).

**Fig. 2:**
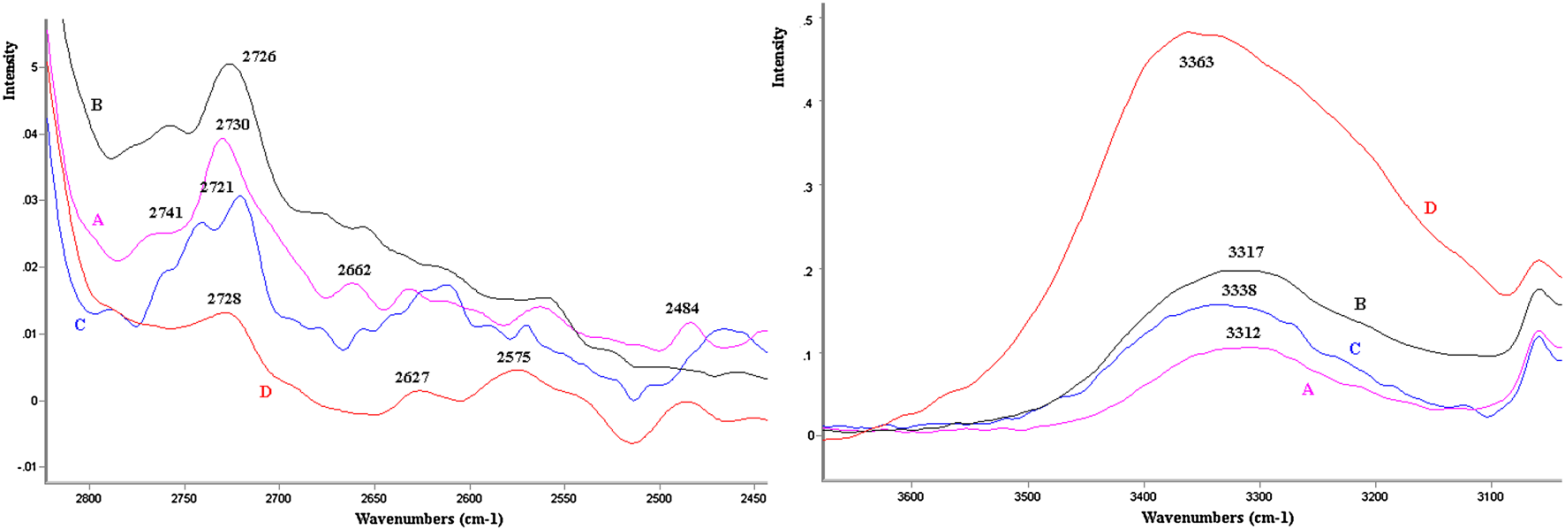
Fragment of FT Raman spectra of the samples: D = AGA-healthy, appropriate for gestational age; A = LGA-healthy, large for gestational age; B = IUGR-intrauterine growth restriction; C = IUGR-G- mothers with gestosis (pregnancy toxemia)

In the case of the AGA tissue—healthy, appropriate for gestational age (Fig. 2)—the increase in the intensity of dispersion and transfer was observed within the frequency of 3425 cm^−1^*ν*_as_ (O–H)–3300 cm^−1^*ν*_as_ (O–H) which indicates the dynamics of interaction in the scope of hydrogen bonds in healthy tissues. While within the frequency of 3500–2500 cm^−1^*ν*(O–H),*ν*(=CH), *ν*(CH_3_), *ν*(CH_2_) shifts related to the presence of lipids in tissues are observed (Fig. 2). It can be postulated that this range, in detailed studies, may prove useful with regard to metabolic and transport dysfunctions of the placenta.

The issue of the so-called biological tissues fluidity is of particular importance in the process of proper oxygenation of tissues. Previously, the dynamics of conformational changes in the lipid acyl chains was analyzed in model membranes [9]. A distinctive feature of the transition from the gel phase to the liquid crystalline phase is frequency upshifting and band broadening. The reason for the increase in frequency and the broadening of the bands is generally the diminution of the conformational order of the lipid acyl chains as well as growth of their dynamics [10, 11]. It cannot be precluded that in pathologically altered placentas, as in preeclampsia, hypoxia of trophoblast cells can be found. As a consequence, oxidative stress and apoptosis of placenta cells occur. In order to determine oxidative stress levels [12], the spectroscopic measurement of peroxides in tissue samples may be used. It is also worth noting that oxidative stress manifested by lipid and protein oxidation among other indices is observed in most of the neurodegenerative and neurotoxic disorders, both as a cause and a consequence of the pathogenic pathways. In a previous article [13], it was suggested that ROS generated by amphetamine-mediated oxidative stress can induce formation of β-sheet rich proteins, which can be of amyloid β-like character.

The *ν*_OH_ band of hydroperoxides located in the 3500–3000 cm^−1^ region and the amide A/amide B ratio can give a screening test in diagnosis and prediction of women who are at great risk to develop preeclampsia [14]. However, it should be added that this region, the CH stretching bands (1750–1700 cm^−1^) alike, is masked by the analogous band of water. The region (1649–1659 cm^−1^) is traditionally assigned to the structure of α-helix [15], whereas the bands in the range 1620–1680 cm^−1^ to the so-called β-sheet aggregates [16]. On the other hand, amide bands II and III (1600–1400 cm^−1^) undergo a shift depending on modification processing of the samples.

**Fig. 3:**
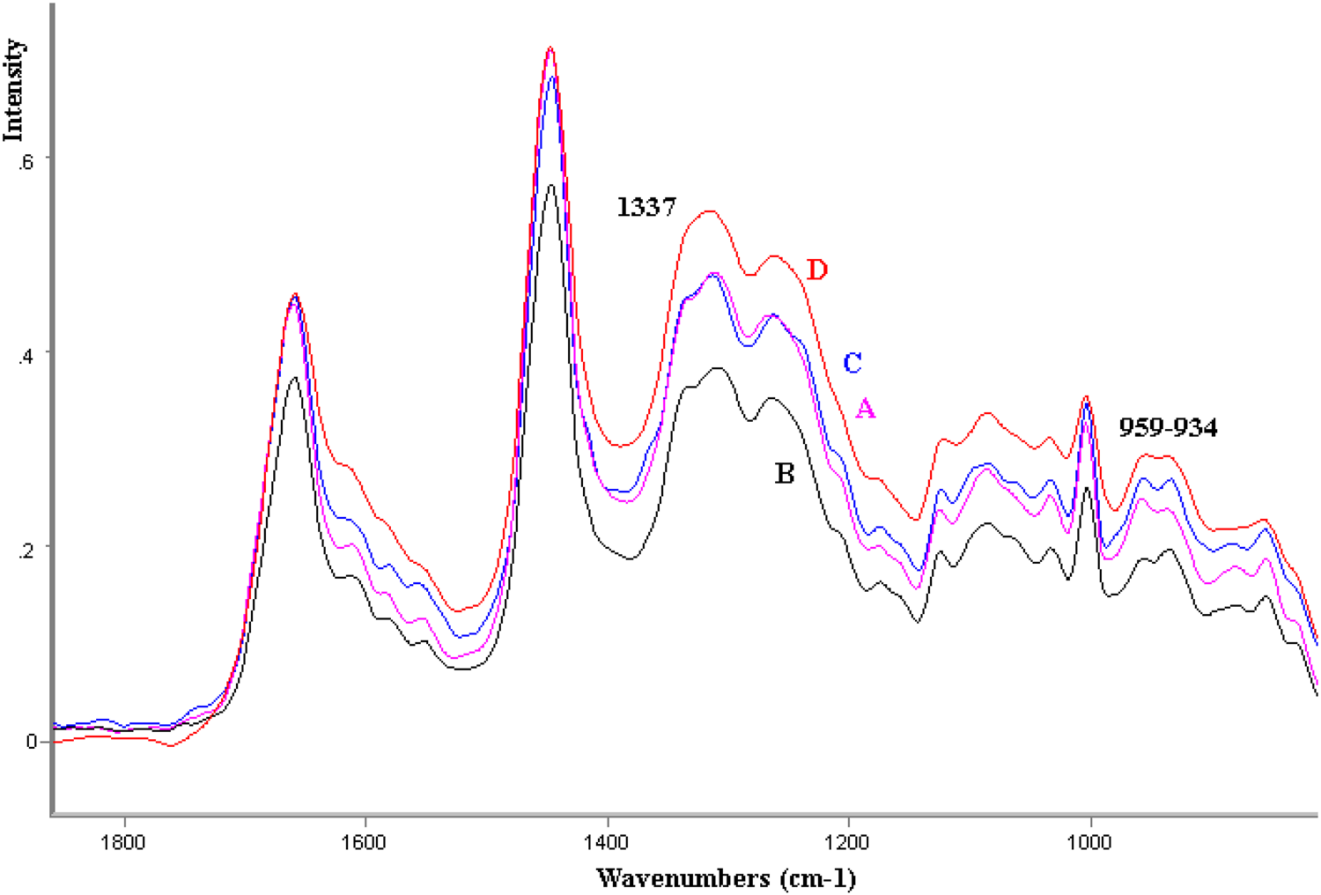
Fragment (1800–800 cm^−1^) of FT Raman spectra of the samples: The average FTR spectra: D = AGA-healthy, appropriate for gestational age; A = LGA-healthy, large for gestational age; B = IUGR-intrauterine growth restriction; C = IUGR-G- mothers with gestosis (pregnancy toxemia); A4a and C6a exemplary samples

In this study, in the average spectra, the lack of interference in this region for healthy tissues was noted. In this respect, the study demonstrates shifts within the band from lipid esters and modification of amide I and II bands (1700–1500 cm^−1^), particularly amide III bands (1418–1200 cm^−1^) for pathological tissues (Fig. 3).

In the Raman spectra research (Fig. 3), the area of lipid interactions 1334–1317 cm^−1^ was exposed for healthy tissues compared to pathological tissues where an evident decrease of intensity of these bands is observed.

The authors’ previous study [6] on burn human skin indicates specific sensitivity of collagen at the molecular level. Post-modifying shifts of wavenumbers in the whole range of 1280–1245 cm^−1^ (characteristic plateau in intact allogeneic skin) prove regeneration of tissues in the course of modification by antioxidants.

**Fig. 4:**
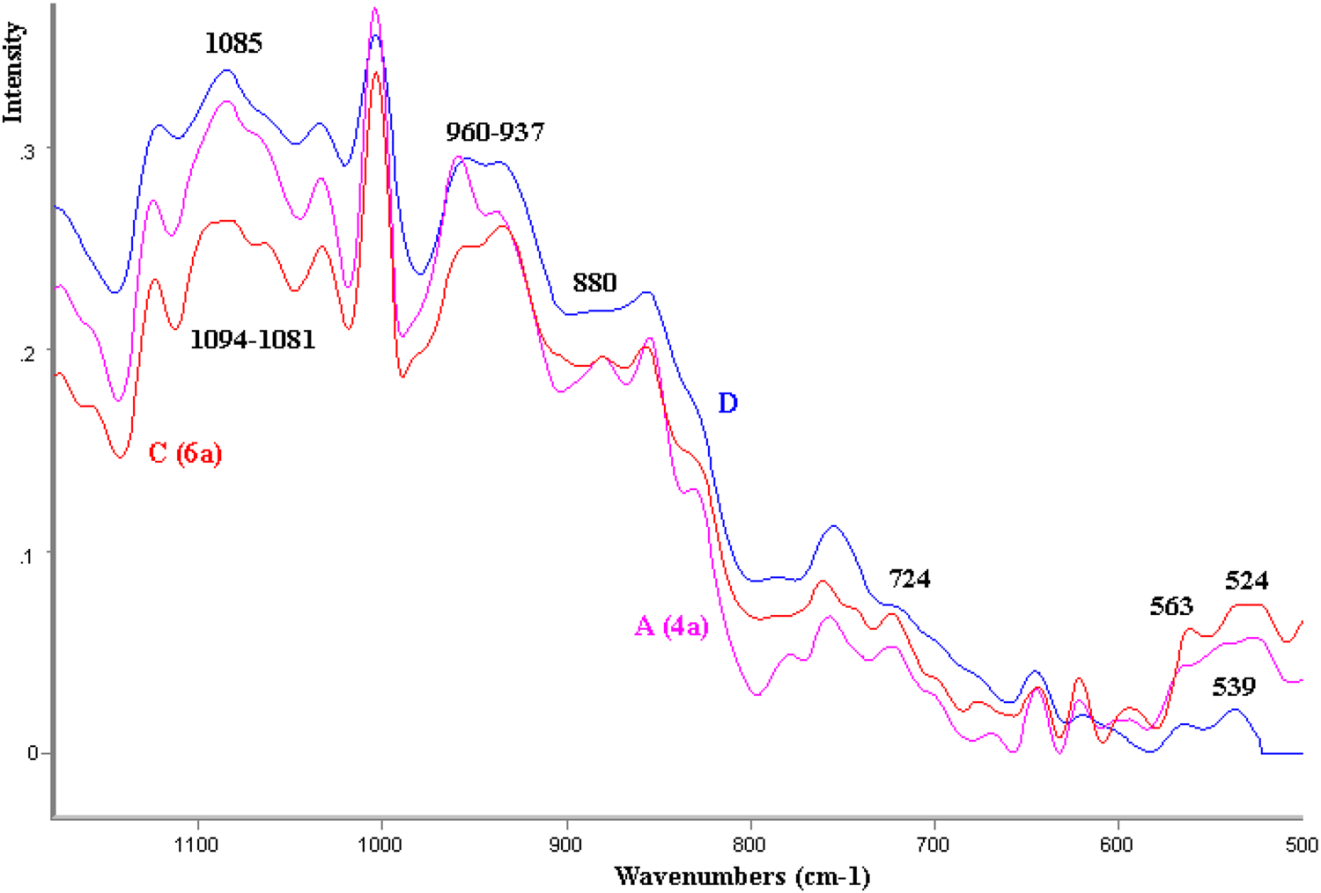
Fragment (1150–500 cm^−1^) of FT Raman spectra of the samples: D-healthy, appropriate for gestational age; A(4a)-healthy, large for gestational age; C(4a)-mothers with gestosis (pregnancy toxemia); A4a and C6a exemplary samples

In this study (Fig. 3), the placental tissue is characterized by variability in the frequency of 1085–880 cm^−1^; the band that originated from sugar-chain was at around 1085 cm^−1^ (originating from the C–OH band) [17]. The glycan-related band in the IUGR placenta [18] may be observed at 1081 cm^−1^. The amide I and III bands, a band in the 890–945 cm^−1^ region is characteristic of an α-helical keratin conformation [19]. In this study on average spectra, plateau within the frequency of 950–933 cm^−1^ and 885 cm^−1^ is demonstrated but, at the same time, in the case of selected dried placenta samples, (A4a) increased intensity of the 959 cm^−1^ band was observed along with simultaneous decreased intensity of dispersion in the (C) sample—children with intrauterine growth restriction (IUGR) born by mothers with pregnancy-induced hypertension (C6a) (Fig. 4).

It should be noted that plateau within the frequency of 885 cm^−1^ for healthy tissues indicates finding spectroscopic biomarkers of regular and pathological placental tissue also in this spectral region.

**Fig. 5:**
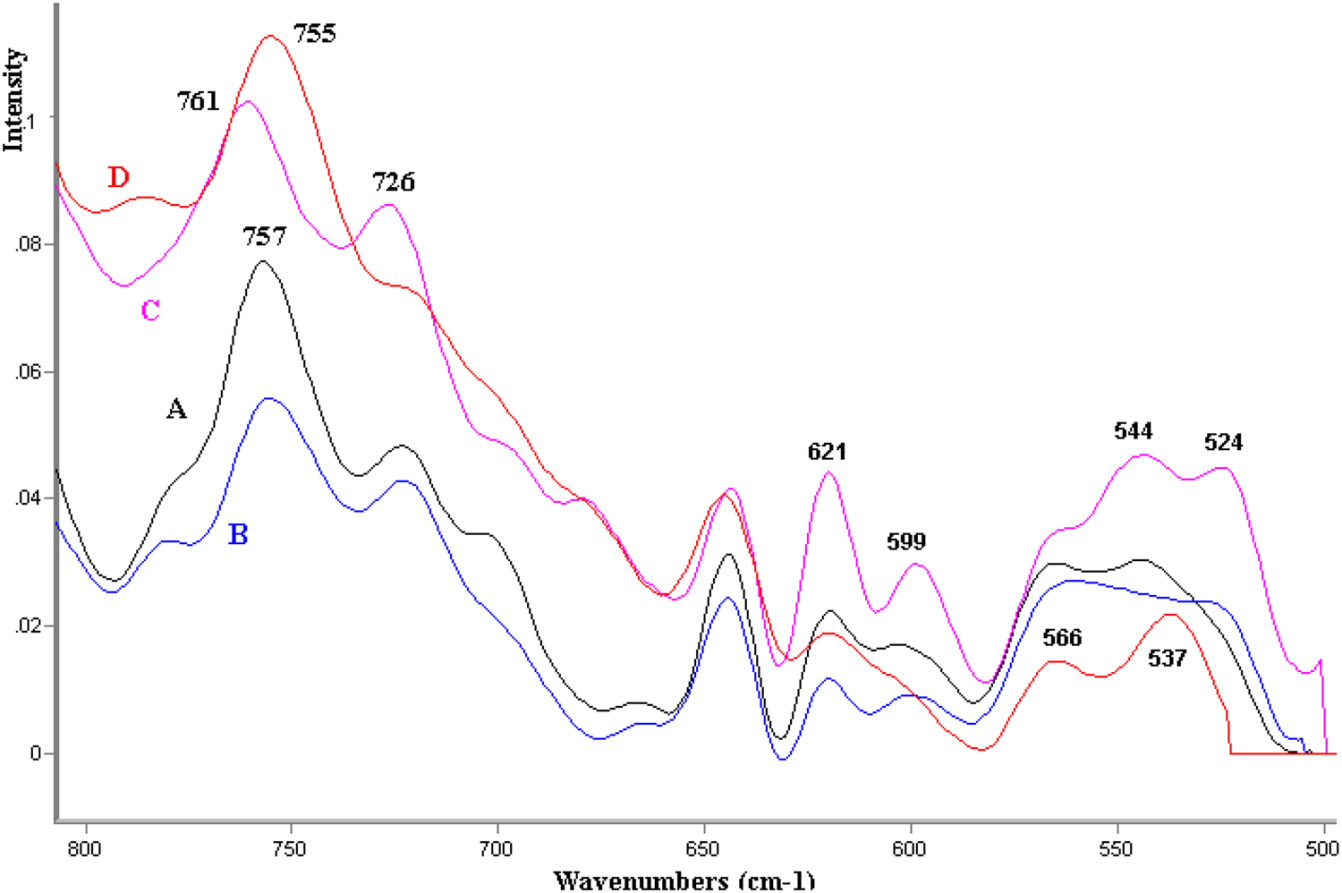
Fragment (800–500 cm^−1^) of FT Raman spectra of the samples: the average FTR spectra: D = AGA-healthy, appropriate for gestational age; A = LGA-healthy, large for gestational age; B = IUGR-intrauterine growth restriction; C = IUGR-G-mothers with gestosis (pregnancy toxemia)

This study also demonstrated shifts within frequencies characteristic of [20, 21] methionine, cysteine, and cystine in average spectra (750–500 cm^−1^) (Fig. 5). For example, for the *trans* form of methionine, the C-S stretching vibrational bands in pathological tissues IUGR-G appear at 620 and 726 cm^−1^. Evident conformational changes within the disulphide bonds 540 cm^−1^ (S–S trans-gauche-trans), 525 (S–S gauche-gauche-trans) are also observed. Among protein side-chain interactions, the disulphide bond is particularly important because it gives additional stability to the folding of a protein. Identified changes within the S–S bonds differentiating healthy tissues from pathological ones indicate finding spectroscopic biomarkers also in this spectral region.

In the presented paper, the method of LOI flammability testing was used in an unconventional way. The selection of LOI method for this type of research was based on similar research on protein fibers (e.g., wool) [22]. Basically, the LOI results for human skin are varied; they are different in terms of the type of skin damage (e.g., thermal or electric burn), the type of tissue: xenogeneic unburned skin, allogenic unburned full-thickness skin, burned human skin [23]. The results shown in work [2, 3] point to an increase in the LOI for samples treated with a mixture of antioxidants and a decrease in the LOI for samples treated with a mixture of antioxidants and DMSO solutions.

Whereas this study found that lower LOI values occur in the case of samples less supplied with blood and higher values in the more supplied with blood ones; Higher LOI values in pathological tissues: 22.7–22.8%, lower in regular, healthy tissues: 21.8%.

**Fig. 6:**
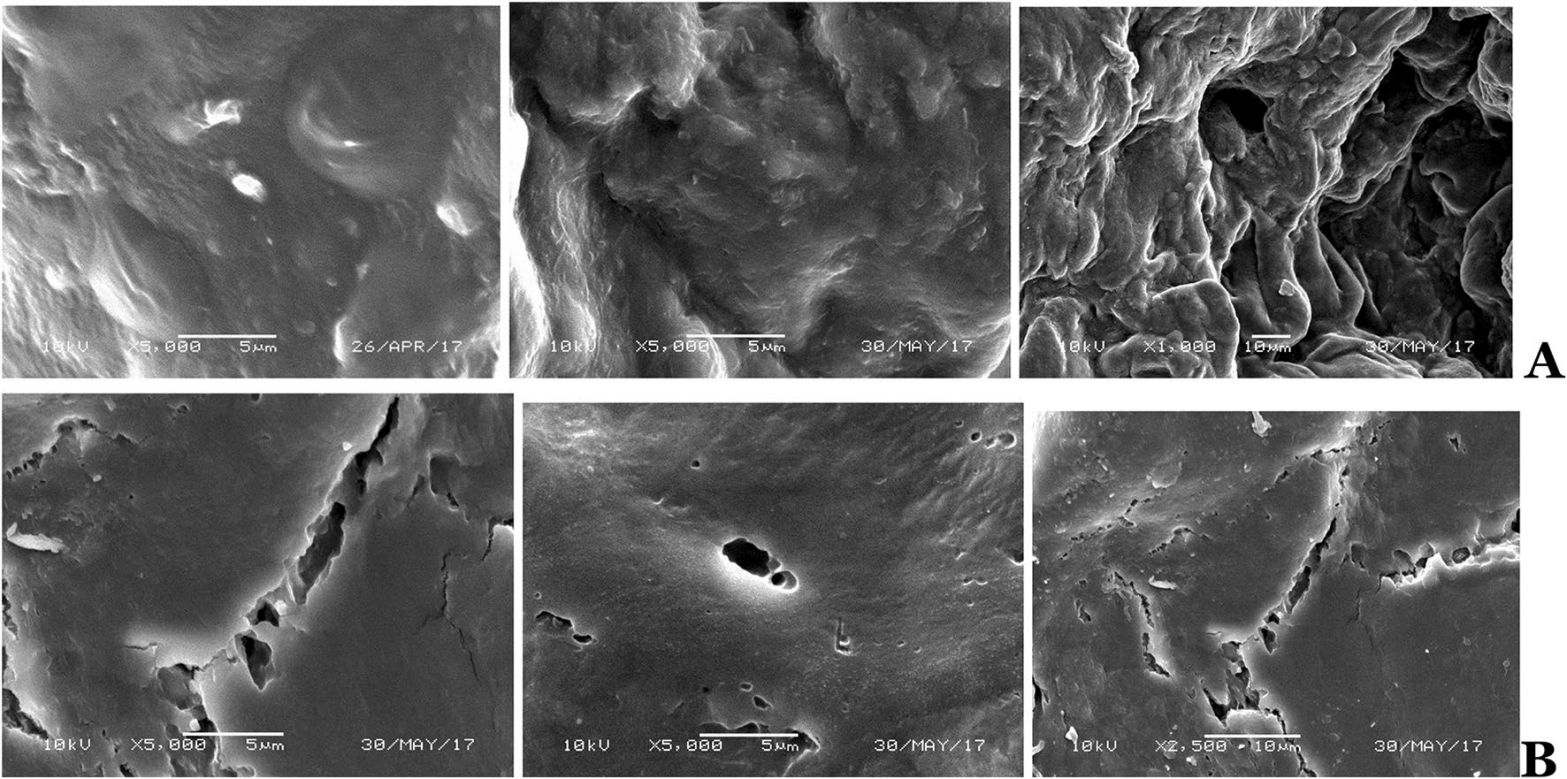
Illustration of the surface of placental tissues acquired using S.E.M. technique (×5000 or ×2500; ×1000). **a** Healthy, **b** pathologically altered placentas

An illustration of the above research is Fig. 6 which differentiates surface of the healthy placental tissue with clearly homogeneous topography from the tissue of pathologically altered placentas, defragmented, and damaged.

One of the key elements that influence proper fetal development is adequate intrauterine nutrition, i.e., providing the developing child with the right amount of building and energetic components as well as with many other micro- and macromolecules. Nutrition of the fetus is a highly complicated process which depends on the functioning of the maternal-placental-fetal metabolism involving all substances necessary for the fetus. Nutrients essential for the development of the baby in the prenatal and postnatal period include different compounds like fatty acids and others.

Structural research will give basis for a deeper analysis of metabolic processes taking place at the junction of the mother’s and child’s organisms, and particularly for the assessment of intrauterine nutrition. Combination of the structural research with the assessment of, for example, maternal-placental-fetal metabolism in mothers who gave birth to IUGR babies will allow for a better understanding of the still very unclear issues related to metabolism at the junction of the organisms of the mother and child, and especially those concerning the relation between fatty acids, carbohydrates, proteins, active peptides etc.

The result of this study will be used in further research on maternal-placental-fetal metabolism to, for example, provide a comprehensive explanation of the phenomenon known as “Biomagnification” or to answer the questions of whether the changes of molecular structure observed in the FTIR and FTR spectra point to the efficiency of fatty acid transport through placenta membranes and explain how the disorders in maternal-placental-fetal metabolism affect the later process of milk biosynthesis modifying its composition compared to the composition of the milk of mothers who gave birth to AGA children. Subjects participating in the study were women with varying degrees of pregnancy pathology, such as SGA, LGA, IUGR, and IUGR-G. To examine the above issues in more detail, research should be extended to include fetal hypotrophy, varying degrees of prematurity, and other common pathologies.

## Conclusions

Changes in the molecular structure observed in the FTR spectra in this study may be a spectroscopic image of placenta functions that is an indicator of efficiency of transport controlled by syncytiotrophoblast in the course of various pathologies. They also document a complex goal of our research that is finding spectroscopic biomarkers of regular and pathological placental tissue.
